# Occipital nerve stimulation for refractory facial pain following occipital pain onset: a case report supporting cervicotrigeminal convergence

**DOI:** 10.3389/fpain.2026.1917660

**Published:** 2026-08-18

**Authors:** Marina Raguž, Marko Tarle, Koraljka Hat, Petar Marčinković, Sven Krušić, Tonko Marinović, Darko Chudy

**Affiliations:** 1 Department of Neurosurgery, Dubrava University Hospital, Zagreb, Croatia; 2 School of Medicine, Catholic University of Croatia, Zagreb, Croatia; 3 Department of Maxillofacial Surgery, Dubrava University Hospital, Zagreb, Croatia; 4 School of Dental Medicine, University of Zagreb, Zagreb, Croatia; 5 Medicine of Sports and Exercise Chair, Faculty of Kinesiology, University of Zagreb, Zagreb, Croatia; 6 School of Medicine, University of Zagreb, Zagreb, Croatia

**Keywords:** cervicotrigeminal convergence, greater occipital nerve, neuromodulation, occipital nerve stimulation, refractory facial pain, trigeminocervical complex

## Abstract

**Introduction:**

Cervicotrigeminal convergence within the trigeminocervical complex (TCC) provides a neuroanatomical basis for referral of nociceptive input from upper cervical structures to trigeminal territories. However, clinical evidence linking occipital pain onset, trigeminal symptom propagation, and successful treatment with occipital nerve stimulation (ONS) remains limited.

**Case presentation:**

A 35-year-old woman developed persistent right-sided occipital, retroauricular, and upper cervical pain approximately eight years before presentation, which progressively spread into trigeminal territories. Multiple pharmacological therapies, greater occipital nerve neurolysis, microvascular decompression, and treatments targeting temporomandibular dysfunction failed to provide sustained relief. Following multidisciplinary evaluation, diagnostic greater occipital nerve (GON) blockade resulted in complete temporary resolution of pain. The patient subsequently underwent bilateral ONS, resulting in marked pain reduction (VAS 8–10/10 to 2/10), contraction of the pain distribution, discontinuation of opioid-containing analgesics, and sustained clinical improvement during follow-up.

**Conclusion:**

This case supports cervicotrigeminal convergence as a plausible mechanism underlying refractory facial pain following occipital pain onset. Complete temporary pain relief after diagnostic GON blockade and sustained improvement following ONS suggest that occipital neuromodulation may represent a therapeutic option in carefully selected patients.

## Introduction

Facial pain is traditionally interpreted as a disorder originating within trigeminal pathways. Consequently, diagnostic evaluation and treatment strategies are commonly directed toward trigeminal, dental, temporomandibular, otorhinolaryngological, or maxillofacial etiologies. However, not all facial pain originates within the trigeminal system. In selected patients, pain perceived within trigeminal territories may arise from structures outside the trigeminal distribution, resulting in diagnostic uncertainty, ineffective treatments, and substantial patient morbidity (1, 2).

A neuroanatomical basis for this phenomenon is provided by the cervicotrigeminal complex (CTC), historically referred to as the trigeminocervical complex. Within this network, nociceptive afferents from the trigeminal nerve and upper cervical roots converge onto shared second-order neurons in the spinal trigeminal nucleus caudalis and upper cervical dorsal horns, creating the substrate for bidirectional referral of pain between trigeminal and cervical territories (3–6). Consequently, nociceptive input originating from upper cervical structures may be perceived as pain within orbital, maxillary, mandibular, auricular, or diffuse hemifacial regions, whereas trigeminal pathology may manifest with occipital or cervical symptoms (4–7).

Among upper cervical pain syndromes, occipital neuralgia and occipital neuropathic pain are particularly relevant because occipital nociceptive input may extend beyond the posterior scalp through CTC-mediated referral mechanisms. Clinical observations have demonstrated trigeminal facial pain secondary to greater occipital nerve pathology, supporting the concept that cervical afferents can serve as dominant drivers of symptoms perceived within trigeminal territories (7, 8). More recently, the CTC has emerged as a unifying framework for understanding refractory neuropathic pain syndromes involving the face, occipital region, and upper cervical spine (9).

These concepts are particularly relevant when considering neuromodulation. If cervical nociceptive input contributes to the generation or maintenance of facial pain, modulation of occipital afferents should theoretically influence trigeminal symptoms. Peripheral nerve stimulation has demonstrated promising results in trigeminal neuropathic pain and persistent idiopathic facial pain (10–12), while occipital nerve stimulation (ONS) is an established treatment for occipital neuralgia and selected medically refractory headache disorders (13, 14). Emerging evidence suggests that ONS may exert therapeutic effects beyond the occipital territory through modulation of shared cervicotrigeminal pathways. Notably, successful treatment of refractory trigeminal neuralgia using ONS has been reported despite the absence of direct trigeminal stimulation (15).

Recent multidisciplinary approaches have further emphasized the importance of phenotype-based assessment and identification of dominant nociceptive generators in patients with refractory craniofacial pain (16). Nevertheless, clinical reports documenting the complete sequence of occipital pain onset, subsequent spread into trigeminal territories, diagnostic response to greater occipital nerve blockade, and successful treatment through occipital neuromodulation remain scarce.

Here, we report a patient whose pain originated in the occipital, retroauricular, and upper cervical regions before progressively spreading into trigeminal territories over several years. Following failure of multiple pharmacological, maxillofacial, and neurosurgical interventions, complete temporary pain relief after greater occipital nerve blockade led to bilateral occipital nerve stimulation, resulting in marked and sustained clinical improvement. This case provides functional clinical evidence supporting cervicotrigeminal convergence and highlights the potential role of occipital neuromodulation in carefully selected patients with refractory facial pain following occipital pain onset.

## Case presentation

### Patient information

A 35-year-old Caucasian woman, employed as a primary school teacher, married with two children, was referred to the Multidisciplinary Team for the Management of Refractory Head and Neck Pain Disorders at Dubrava University Hospital, Zagreb, Croatia, because of severe refractory right-sided facial pain that had persisted for approximately eight years despite extensive pharmacological, surgical, and interventional treatment.

**Figure 1:**
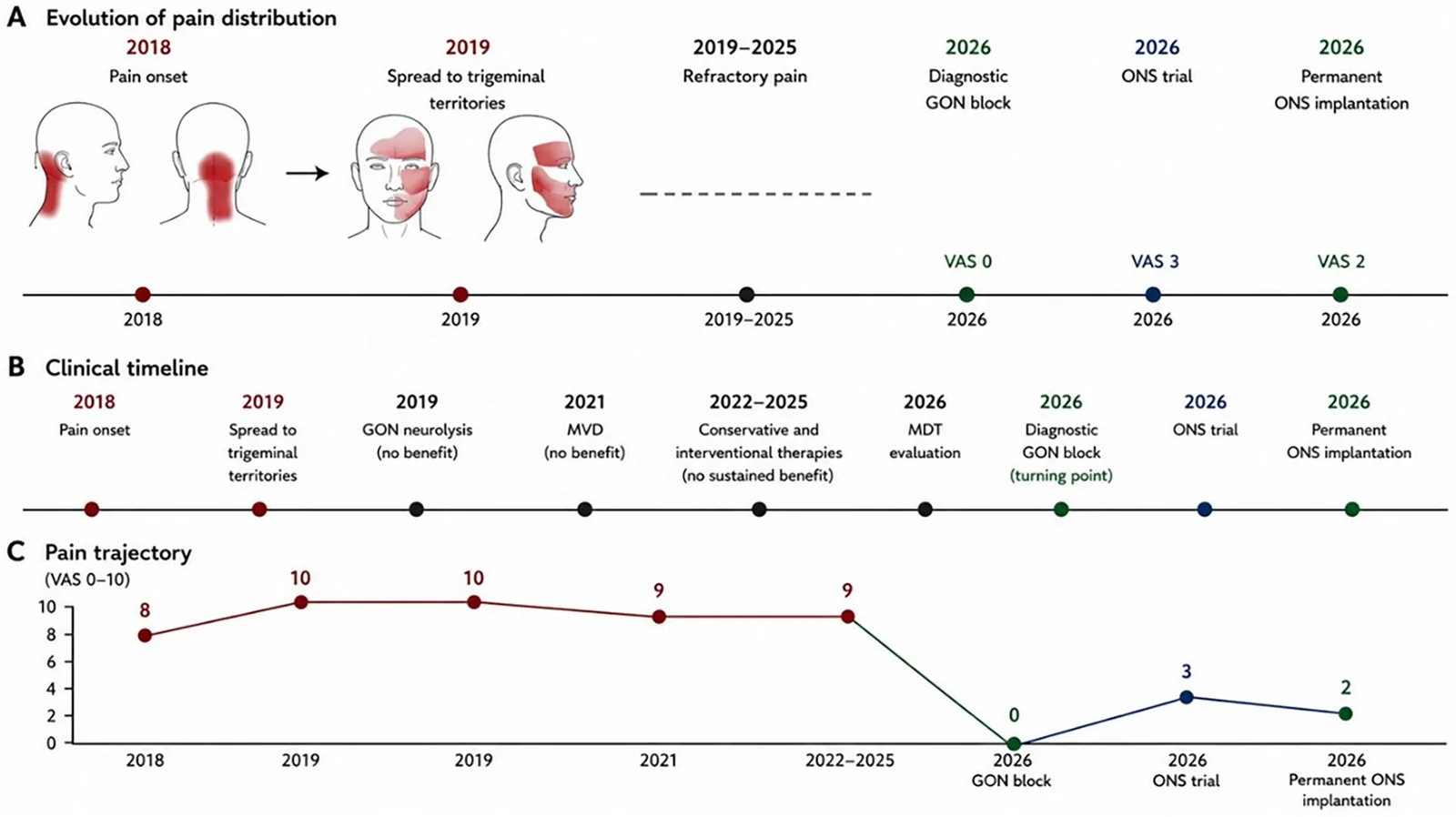
Clinical timeline and pain trajectory during the patient's diagnostic and therapeutic journey. **(A)** Evolution of pain distribution from occipital and upper cervical onset to involvement of trigeminal territories, followed by diagnostic greater occipital nerve (GON) blockade and occipital nerve stimulation (ONS). **(B)** Major clinical events, including unsuccessful pharmacological, interventional, and surgical treatments, multidisciplinary reassessment, and neuromodulation. **(C)** Pain trajectory measured using the visual analogue scale (VAS, 0-10), demonstrating prolonged severe pain, complete temporary relief following diagnostic GON blockade, and sustained improvement after ONS implantation.

Symptoms first developed approximately eight years before presentation as persistent pain localized to the right occipital, retroauricular, and upper cervical regions. The patient denied preceding trauma, infection, dental procedures, or other identifiable triggering events. Initial pain intensity was estimated at 7–8 on a 10-point visual analogue scale (VAS). Over the following year, pain progressively extended into trigeminal territories, involving the supraorbital, infraorbital, preauricular, and mandibular regions. The pain was described as deep, aching, burning, and intermittently stabbing, with occasional electric shock-like sensations affecting the lower face. Symptom intensity gradually increased to VAS 9–10/10. Pain was exacerbated by chewing, speaking, exposure to cold air, wind, and weather changes, resulting in sleep disturbance, impaired daily functioning, and increasing dependence on analgesic medication. The chronological evolution of symptoms and treatments is summarized in Figure 1.

### Clinical findings

**Figure 2:**
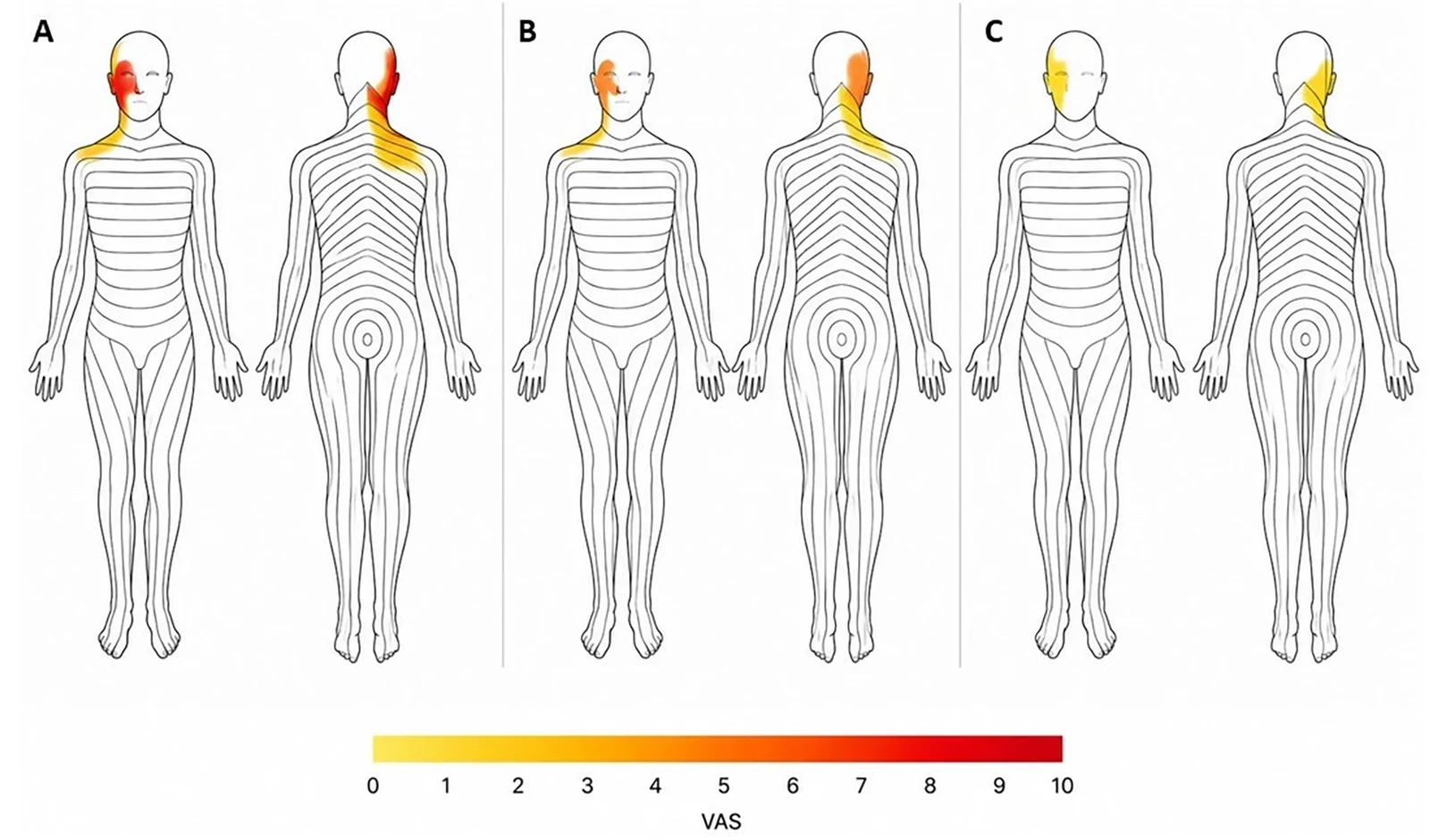
Evolution of patient-reported pain distribution following occipital neuromodulation. **(A)** Preoperative pain distribution. Pain drawing obtained before ONS demonstrating extensive involvement of the right occipital, retroauricular, upper cervical, and trigeminal regions. The highest pain intensity was localized to the right temporal, periorbital, and retroauricular areas, with radiation toward the lateral neck and shoulder. Pain intensity reached VAS 8–10/10 despite multiple previous pharmacological and surgical interventions. **(B)** ONS trial stimulation. Pain drawing obtained during the six-day occipital nerve stimulation trial. A marked reduction in both pain intensity and distribution is evident, particularly within the trigeminal territories. Residual symptoms remained localized primarily to the retroauricular and upper cervical regions, accompanied by a substantial decrease in analgesic requirements. **(C)** Follow-up after permanent ONS implantation. Pain drawing was obtained following permanent bilateral occipital nerve stimulation. Further reduction of pain intensity and affected surface area is observed, with only mild residual discomfort confined to limited retroauricular and upper cervical regions. The patient reported sustained clinical improvement with a reduction in pain intensity to approximately VAS 2/10. Color scale: Patient-reported pain intensity according to the Visual Analogue Scale (VAS), ranging from 0 (yellow) to 10 (red).

At presentation, pain involved the right occipital region, retroauricular area, lateral neck, temporal region, orbit, cheek, preauricular region, and mandible. The distribution extended beyond the territory of a single peripheral nerve and did not conform to a classical trigeminal neuralgia pattern. Neurological examination was unremarkable. The patient was fully alert and oriented, with normal speech and cognition. Pupils were equal and reactive to light, ocular movements were full, and no diplopia or nystagmus was present. Facial motor function was symmetrical, facial sensation was preserved, and the remaining cranial nerves were intact. No focal neurological deficits, cerebellar signs, pyramidal tract signs, or gait abnormalities were identified; muscle strength, deep tendon reflexes, coordination, tandem gait, and Romberg testing were normal. Examination of the temporomandibular joints and masticatory musculature revealed only mild tenderness. Baseline pain intensity was VAS 8/10. Neuropathic pain screening using the DN4 questionnaire yielded a score of 5/10. Pain drawings demonstrated simultaneous involvement of occipital, cervical, and trigeminal territories (Figure 2A), supporting the clinical suspicion of cervicotrigeminal involvement.

### Diagnostic assessment

Prior to referral, the patient underwent extensive diagnostic evaluation and multiple treatment attempts. Initial pharmacological management included pregabalin, titrated from 75 mg twice daily to 150 mg twice daily, carbamazepine up to 400 mg two to three times daily for approximately two years, and duloxetine increased from 30 mg to 60 mg daily. Despite dose escalation and prolonged treatment, none of these therapies provided meaningful or sustained pain relief. Progressive symptom severity ultimately required frequent rescue analgesia, including tramadol/paracetamol and dexketoprofen/tramadol, reaching up to 5–6 tablets daily before multidisciplinary evaluation. Because of the prominent occipital component, right-sided greater occipital nerve pathology was initially suspected. Approximately one year after symptom onset, the patient underwent surgical neurolysis of the greater occipital nerve (GON) with transection of the third and fourth occipital nerves and reconstruction of the third occipital nerve, without clinical improvement.

As facial symptoms became increasingly prominent, trigeminal neuralgia was considered. Approximately three years after symptom onset, the patient underwent microvascular decompression (MVD) of the right trigeminal nerve, which likewise failed to provide symptom relief. Additional interventions, targeting a possible temporomandibular disorder (TMD), including occlusal splint therapy, botulinum toxin injections into the masticatory muscles, physical therapy, acupuncture, and temporomandibular joint arthrocentesis with lavage, failed to provide sustained clinical improvement. At the time of multidisciplinary evaluation, the patient required approximately 5–6 tramadol tablets daily for symptom control.

The Multidisciplinary Team reviewed the case for the Management of Refractory Head and Neck Pain Disorders, comprising specialists in neurology, neurosurgery, maxillofacial surgery, oral surgery, otorhinolaryngology, neuroradiology, pain medicine, physical medicine and rehabilitation, psychiatry, and psychology. The combination of persistent occipital pain, progressive spread into trigeminal territories, previous failure of trigeminal surgery, and absence of objective neurological deficits prompted reconsideration of the underlying pain mechanism. A landmark-guided diagnostic right GON block was therefore performed using 2 mL of a mixture of lidocaine and bupivacaine. The patient experienced complete resolution of both occipital and trigeminal pain lasting approximately 8–10 h. To confirm reproducibility, the GON block was repeated and produced the same complete but temporary pain relief, representing the first intervention since symptom onset to provide substantial relief and strongly supporting involvement of cervicotrigeminal pathways.

Based on the clinical presentation, extensive multidisciplinary evaluation, exclusion of alternative structural causes, characteristic temporal progression from occipital to trigeminal pain, reproducible complete response to diagnostic GON blockade, and sustained improvement following ONS, the patient's clinical presentation was considered most consistent with a mixed cervicotrigeminal pain phenotype, in which cervicotrigeminal convergence represented the most plausible mechanism underlying symptom propagation. Although such a presentation is not currently recognized as a distinct diagnostic entity within the ICOP or ICHD-3 classifications, the clinical phenotype did not fulfill the diagnostic criteria for classical trigeminal neuralgia, painful trigeminal neuropathy, persistent idiopathic facial pain, occipital neuralgia, or cervicogenic headache alone. TMD was carefully evaluated during the multidisciplinary diagnostic work-up but was ultimately considered unlikely to represent the primary pain disorder, given the overall clinical evaluation and the absence of sustained clinical benefit following multiple TMD-directed interventions. Instead, the overall clinical picture was considered most compatible with a mixed cervicotrigeminal pain phenotype, with cervicotrigeminal convergence providing the most plausible pathophysiological explanation.

### Therapeutic intervention

Given the complete but temporary response to GON blockade, peripheral neuromodulation targeting occipital afferent pathways was considered the most appropriate treatment option. Following multidisciplinary consensus, the patient underwent a 6-day bilateral ONS trial using bilateral Medtronic AnkerStim™ occipital leads connected to an external neurostimulator. Initial stimulation was programmed in cycling mode (10 min ON/20 min OFF), with a pulse width of 200 μs, frequency of 40 Hz, and amplitudes of 3.0 mA (right) and 1.0 mA (left). Successful trial stimulation was defined by clinically meaningful pain reduction (>50%), reduced analgesic consumption, functional improvement, and the patient's willingness to proceed with permanent implantation. During the trial period, the patient reported marked pain reduction, decreased analgesic requirements, and contraction of the symptomatic area on pain drawings. Based on the favorable response, permanent bilateral implantation was performed using a Medtronic Inceptiv™ rechargeable implantable pulse generator without perioperative complications.

### Follow-up and outcomes

Clinical follow-up was performed at 1 and 3 months after permanent ONS implantation. Immediate pain relief achieved during the trial stimulation period was maintained following permanent implantation. Pain intensity decreased from a pre-treatment VAS score of 8–10/10 to approximately 2/10 (Figures 2B,C). Pain distribution was markedly reduced, with substantial improvement in both facial and occipital symptoms. Opioid-containing rescue medication was discontinued, and the patient no longer required the previously reported daily tramadol consumption. At the 1-month follow-up, these improvements were confirmed clinically and remained stable at the 3-month follow-up. Residual symptoms were limited to mild temporomandibular discomfort and occasional masticatory muscle pain. The patient experienced meaningful functional recovery, with reduced analgesic requirements and return to daily activities previously limited by pain.

### Patient perspective

The patient described the years preceding neuromodulation as physically and emotionally exhausting, with persistent pain significantly affecting daily activities, sleep, and social functioning. Following occipital nerve stimulation, she reported substantial improvement in quality of life, reduced reliance on analgesic medication, and a return to activities that had previously been limited by pain. The patient considered the treatment outcome highly satisfactory and expressed that the improvement exceeded her expectations after multiple unsuccessful therapeutic interventions.

## Discussion

This case illustrates a clinically important and often underrecognized mechanism of refractory facial pain: symptoms perceived predominantly within trigeminal territories may be driven by occipital and upper cervical nociceptive input. The significance of this report lies not merely in the successful use of ONS, but in the coherent clinical sequence observed over several years. Pain originated in the occipital and upper cervical regions, progressively spread into trigeminal territories, failed to respond to trigeminal- and maxillofacial-directed interventions, was completely abolished by diagnostic GON blockade, and subsequently improved following chronic occipital neuromodulation. Taken together, these observations provide functional clinical evidence supporting cervicotrigeminal convergence as the most plausible mechanism underlying symptom propagation in this patient.

The CTC, historically referred to as the trigeminocervical complex, provides the principal neuroanatomical substrate explaining this pattern. Since the seminal anatomical observations of Kerr (3), subsequent experimental and clinical studies have consistently demonstrated convergence of trigeminal and upper cervical afferents within a shared nociceptive network (4, 5). The broader functional relevance of cervical afferent input in head and facial pain has also been emphasized by Goadsby and Bartsch (17), who highlighted the central role of trigeminal-cervical interactions in the neurobiology of craniofacial pain. More recently, Bou Malhab et al. (6) described the trigeminal cervical complex as a broader neural network affecting the head and neck, while Raguž et al. (9) proposed the CTC as an integrative hub underlying several refractory neuropathic pain syndromes involving the face, occipital region, and upper cervical spine. Within this framework, progression from occipital pain to involvement of V1, V2, and V3 territories is not anatomically paradoxical but represents a clinically plausible consequence of trigeminal-cervical convergence. Experimental and clinical observations support such referral patterns (7, 8).

The clinical phenotype observed in the present patient was inconsistent with classical trigeminal neuralgia. Diffuse pain distribution, coexistence of cervical and trigeminal symptoms, neuropathic characteristics, persistence after MVD, and complete response to occipital blockade collectively suggested that the principal pain generator was unlikely to be confined to the trigeminal root entry zone. Contemporary classifications and guidelines emphasize that facial pain syndromes comprise a heterogeneous group of disorders with distinct mechanisms and therapeutic implications (2, 18–20). Before attributing the patient's symptoms to cervicotrigeminal convergence, an extensive multidisciplinary diagnostic work-up was performed to exclude alternative structural and secondary causes of trigeminal neuropathic pain. Brain magnetic resonance imaging, neurosurgical, neuroradiological, maxillofacial, otorhinolaryngological, and multidisciplinary evaluations failed to identify a structural lesion or alternative pathology explaining the clinical presentation. Therefore, the proposed mechanism was not based solely on the symptom distribution, but on the combination of negative diagnostic investigations, failure of trigeminal-directed interventions, and the reproducible response to diagnostic greater occipital nerve blockade. Accordingly, the present case is best interpreted as a mixed cervicotrigeminal pain phenotype rather than a classical trigeminal disorder. Existing classifications such as ICOP and ICHD-3 provide important diagnostic frameworks but do not currently include a specific category encompassing the constellation of findings observed in this patient. The principal differential diagnoses included classical trigeminal neuralgia, painful trigeminal neuropathy, persistent idiopathic facial pain, occipital neuralgia, temporomandibular disorder, cervicogenic headache, and secondary structural causes of trigeminal pain, all of which were systematically considered during the multidisciplinary diagnostic work-up. In retrospect, the lack of response to MVD may not represent failure of surgery, but rather incorrect localization of the primary pain generator. Moreover, surgical treatment of occipital nerve pathology has been reported to provide benefit in carefully selected patients (21). However, the absence of improvement following occipital nerve surgery in the present case suggests that the dominant pathological process was no longer confined to a peripheral occipital nerve lesion. Similarly, TMD was carefully considered during the diagnostic work-up because of mild temporomandibular joint and masticatory muscle tenderness. Although the patient underwent several TMD-directed interventions, including occlusal splint therapy, botulinum toxin injections, physical therapy, and temporomandibular joint arthrocentesis with lavage, none resulted in sustained clinical improvement. These findings suggested that, although TMD may have represented a limited coexisting musculoskeletal component, it was unlikely to account for the overall clinical presentation. Notably, following successful occipital nerve stimulation, the residual symptoms were largely confined to mild temporomandibular discomfort and occasional masticatory muscle pain. These residual symptoms were considered more consistent with a concurrent mild temporomandibular musculoskeletal disorder than with the previously widespread cervicotrigeminal pain syndrome, suggesting that the predominant neuropathic pain component had been successfully addressed.

The diagnostic GON block represented the pivotal moment in the patient's diagnostic journey. If facial symptoms had been generated primarily within trigeminal pathways, interruption of occipital afferent input would not be expected to abolish the entire pain syndrome. The opposite was observed. Importantly, the block resulted in complete temporary resolution not only of occipital pain but also of symptoms perceived within trigeminal territories. From a mechanistic perspective, the intervention functioned as a reversible physiological experiment: transient interruption of occipital nociceptive input was accompanied by disappearance of facial pain. Previous studies have highlighted the diagnostic value of occipital nerve blocks in headache and occipital neuralgia syndromes (22–25), but the present case extends this concept by demonstrating their utility in identifying cervicotrigeminally mediated facial pain.

The subsequent success of ONS further strengthened this interpretation. ONS is primarily established for occipital neuralgia and medically refractory headache disorders (13, 14). More broadly, peripheral nerve stimulation has emerged as an established therapeutic strategy for selected neuropathic pain syndromes (26) and is increasingly recognized as an important component of contemporary neuromodulation-based pain management (27). However, accumulating evidence suggests that modulation of occipital afferents may influence pain processing beyond the occipital territory. Successful treatment of trigeminal pain using ONS has previously been reported by Fontaine et al. (15). More recently, Balossier et al. demonstrated favorable outcomes of ONS in patients with recurrent trigeminal neuralgia even in the absence of concomitant occipital pain, further supporting the concept that occipital neuromodulation may influence trigeminal pain through shared cervicotrigeminal mechanisms (28). Peripheral nerve stimulation more broadly has demonstrated promising results in trigeminal neuropathic pain and persistent idiopathic facial pain (10–12). The present case extends these observations by demonstrating improvement of trigeminally distributed pain through stimulation of a presumed cervical nociceptive driver. Rather than acting solely through local effects at the site of stimulation, ONS may exert its therapeutic benefit through modulation of shared cervicotrigeminal pain networks.

From a broader neurobiological perspective, this case supports a network-based rather than nerve-based model of chronic craniofacial pain. Repeated nociceptive input from upper cervical afferents may induce central sensitization within the CTC, resulting in expansion of receptive fields and spread of symptoms across multiple craniofacial regions (29). Particularly informative were the serial pain drawings, which demonstrated progressive contraction of the symptomatic area following ONS. Such changes are more consistent with modulation of a sensitized nociceptive network than with treatment of a single peripheral pain source. Similar principles have been proposed for epidural motor cortex stimulation in refractory neuropathic pain (30) and were recently supported by long-term outcome data from our institution (31). Collectively, these observations support the concept that neuromodulation may achieve therapeutic benefit through modification of distributed pain-processing networks rather than solely through local effects at the site of stimulation.

The present case also highlights the value of multidisciplinary evaluation. Prior to referral, multiple interventions targeted plausible but incomplete explanations for the patient's symptoms. Evaluation within a dedicated multidisciplinary team transformed a fragmented diagnostic history into a coherent mechanistic hypothesis that ultimately guided successful treatment, supporting recently proposed phenotype-based approaches to refractory facial pain (16).

### Limitations

This report has several limitations. As a single-case report, it cannot establish causality or determine the generalizability of the proposed pathophysiological mechanism. Although the temporal sequence of occipital pain onset, complete response to diagnostic GON blockade, failure of multiple conventional therapies, and sustained improvement following ONS strongly supports cervicotrigeminal involvement, the proposed mechanism should be regarded as a biologically plausible interpretation rather than definitive proof. Although the diagnostic GON block produced a reproducible complete response, the diagnostic specificity of this finding remains limited and should be interpreted within the context of the overall clinical presentation and multidisciplinary assessment. Furthermore, standardized longitudinal patient-reported outcome measures and validated quality-of-life assessments were not available. Future studies involving larger patient cohorts are needed to validate these observations and further define the role of occipital neuromodulation in cervicotrigeminally mediated facial pain.

## Conclusion

This case highlights the potential role of cervicotrigeminal convergence in the pathophysiology of refractory facial pain. The characteristic progression from occipital and upper cervical pain to trigeminal symptomatology, complete temporary resolution following diagnostic greater occipital nerve blockade, and sustained improvement after occipital nerve stimulation collectively support the hypothesis that occipital afferent pathways may drive pain perceived within trigeminal territories. These findings provide functional clinical evidence supporting cervicotrigeminal convergence as a biologically plausible mechanism underlying symptom propagation in this patient.

Although conclusions are inherently limited by the single-case design, this report highlights the potential value of multidisciplinary evaluation and targeted occipital interventions in carefully selected patients.

## Data Availability

The raw data supporting the conclusions of this article will be made available by the authors, without undue reservation.
